# Supplementary material for: Cuminal Inhibits Trichothecium roseum Growth by Triggering Cell Starvation: Transcriptome and Proteome Analysis
Source: Microorganisms. 2020 Feb 14;8(2):256. doi: 10.3390/microorganisms8020256 (PMC7074788; doi:10.3390/microorganisms8020256)
Supplement: Supplementary file 1 [file microorganisms-08-00256-s001.zip › Supplementary files/Supplementary Figures.docx]

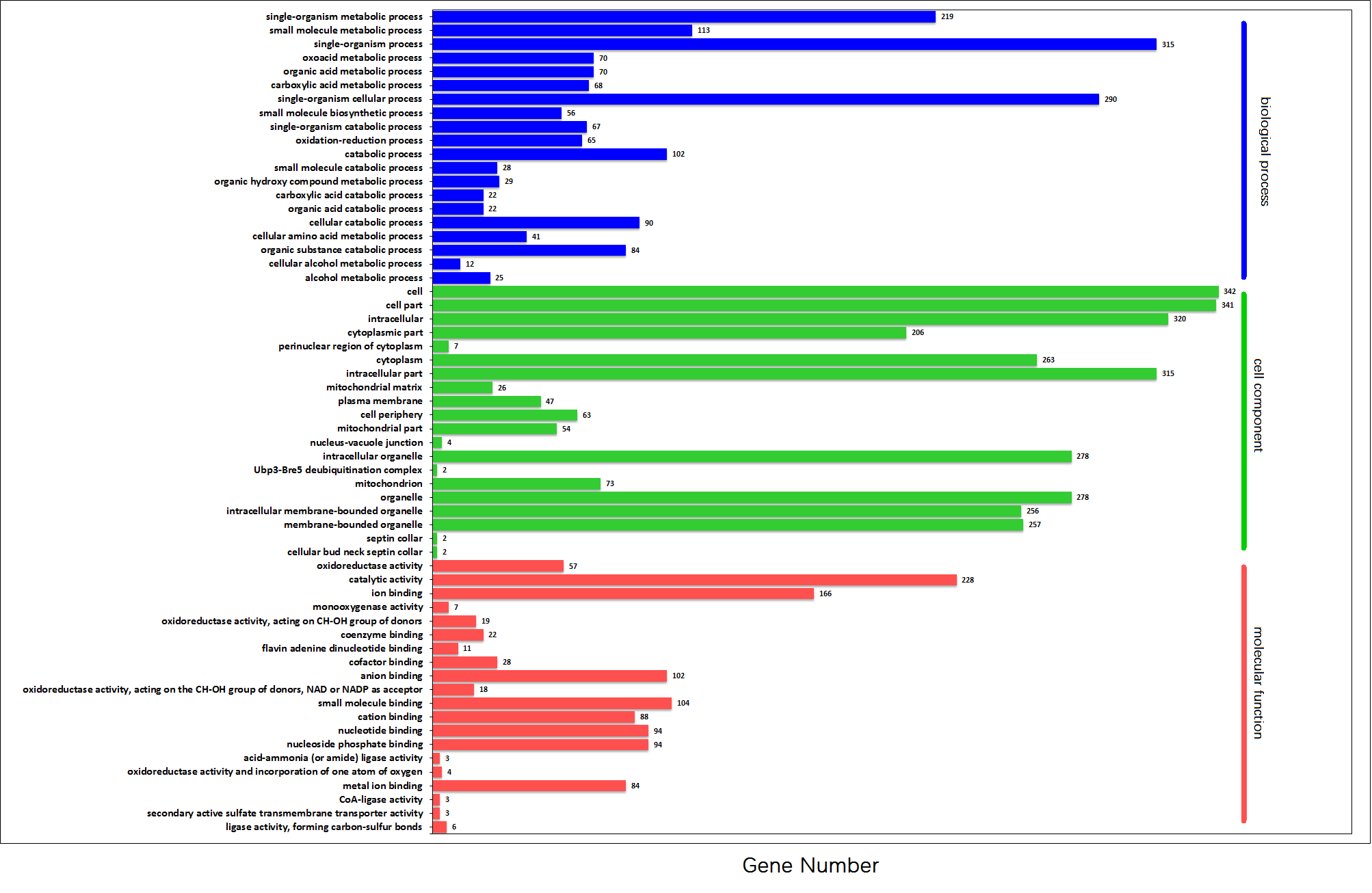


**Figure S1.** GO functional annotation histogram of the up DEGs. The ordinate coordinates represent three GO categories under the level of the GO term and the annotation of the term; the abscissa is the number of up DEGs involved in corresponding terms. Three basic categories of GO annotations include (from top to bottom) biological processes, cellular component, and molecular function.


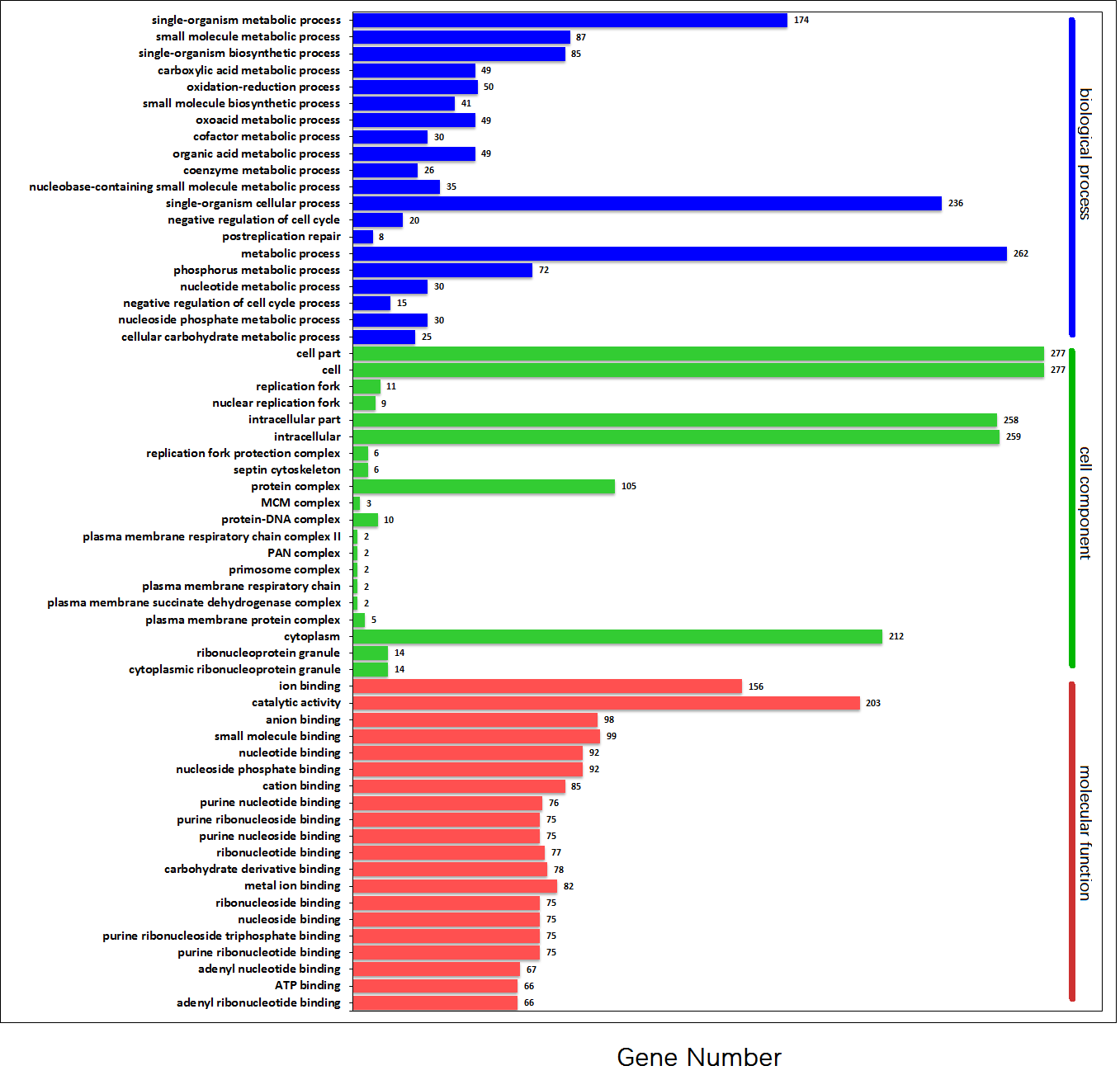


**Figure S2.** GO functional annotation histogram of the down DEGs. The ordinate coordinates represent three GO categories under the level of the GO term and the annotation of the term; the abscissa is the number of down DEGs involved in corresponding terms. Three basic categories of GO annotations include (from top to bottom) biological processes, cellular component, and molecular function.


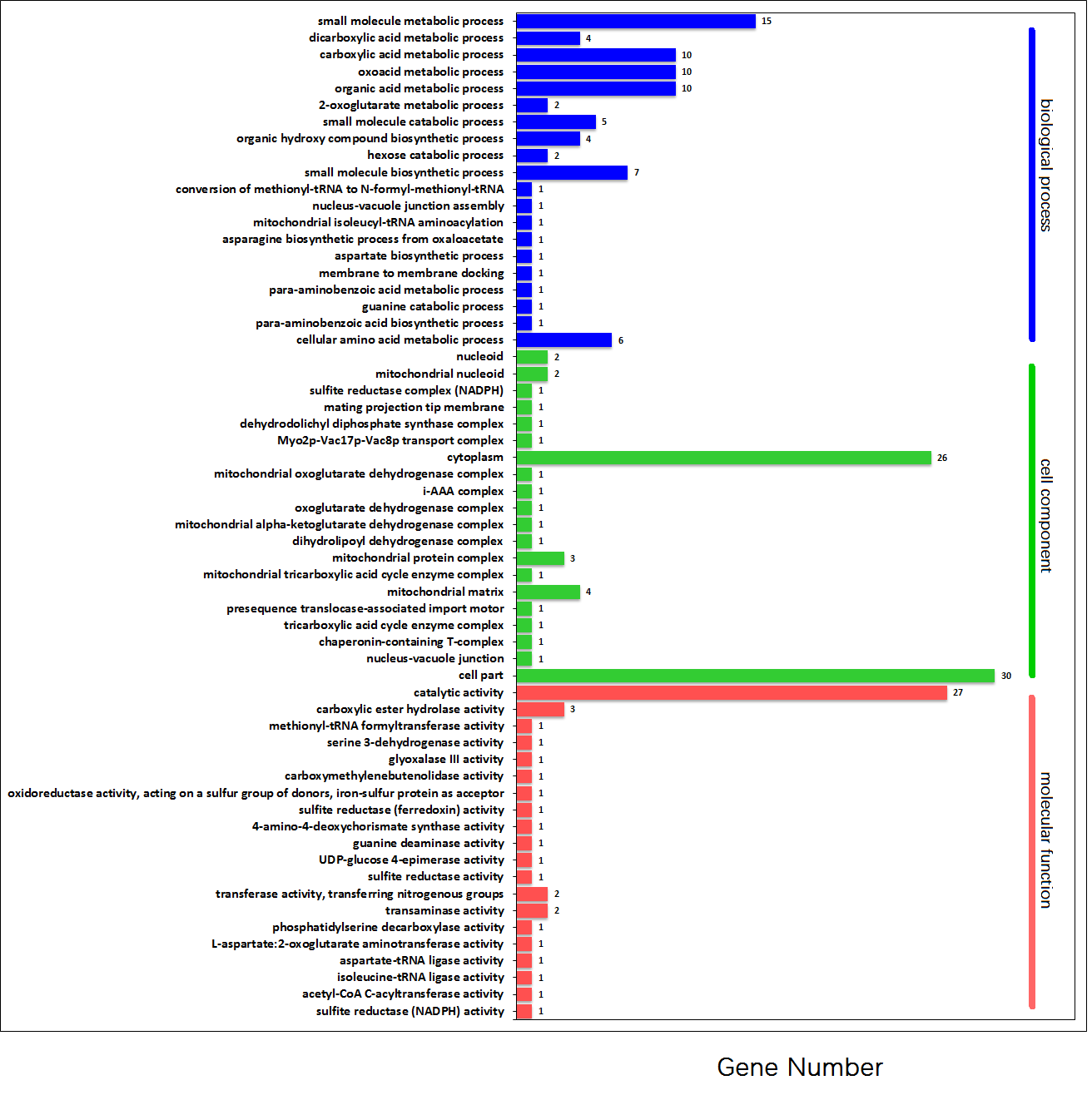


**Figure S3.** GO functional annotation histogram of the up DEPs. The ordinate coordinates represent three GO categories under the level of the GO term and the annotation of the term; the abscissa is the number up DEPs involved in corresponding terms. Three basic categories of GO annotations include (from top to bottom) biological processes, cellular component, and molecular function.


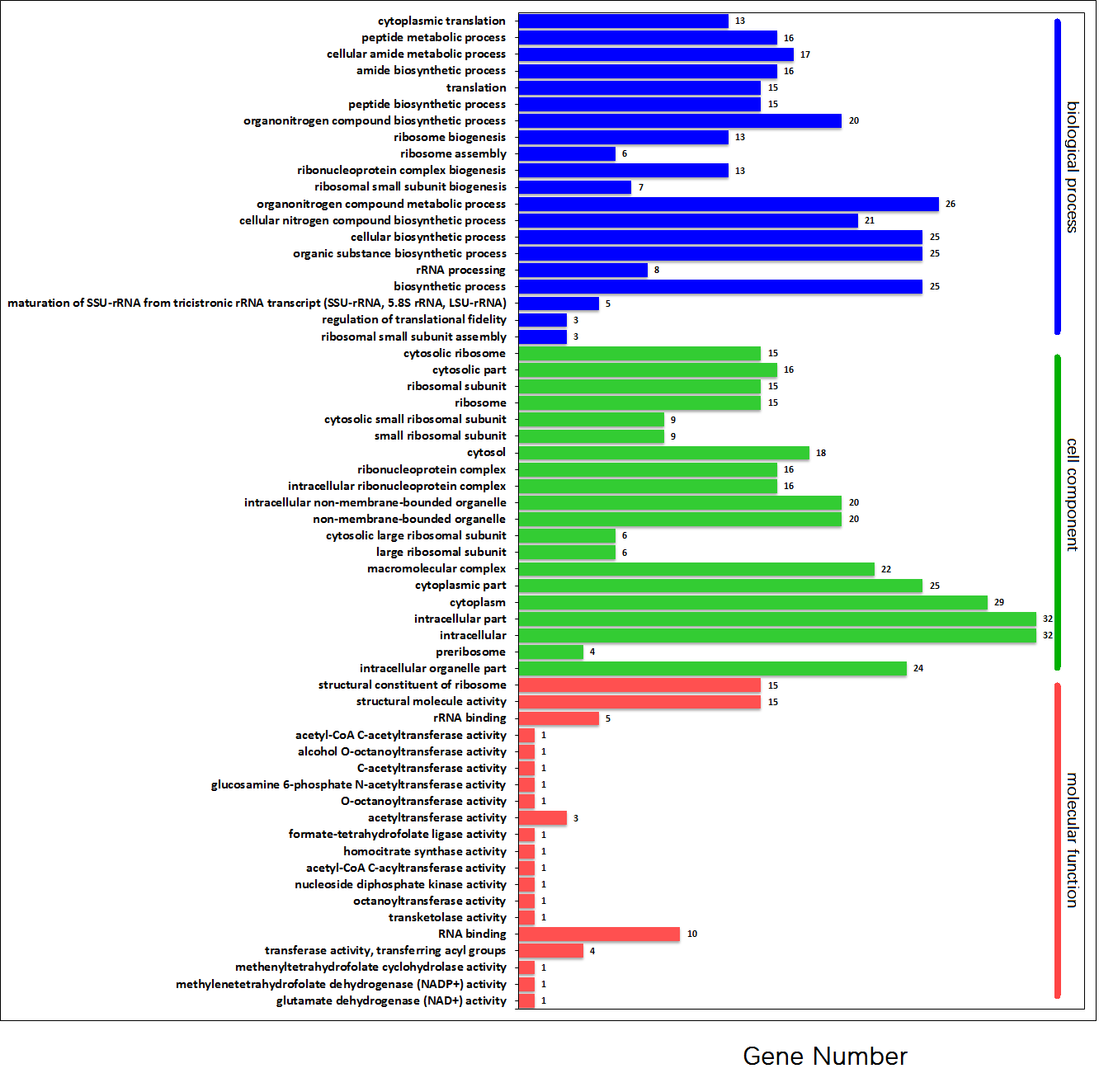


**Figure S4.** GO functional annotation histogram of the down DEPs. The ordinate coordinates represent three GO categories under the level of the GO term and the annotation of the term; the abscissa is the number of down DEPs involved in corresponding terms. Three basic categories of GO annotations include (from top to bottom) biological processes, cellular component, and molecular function.


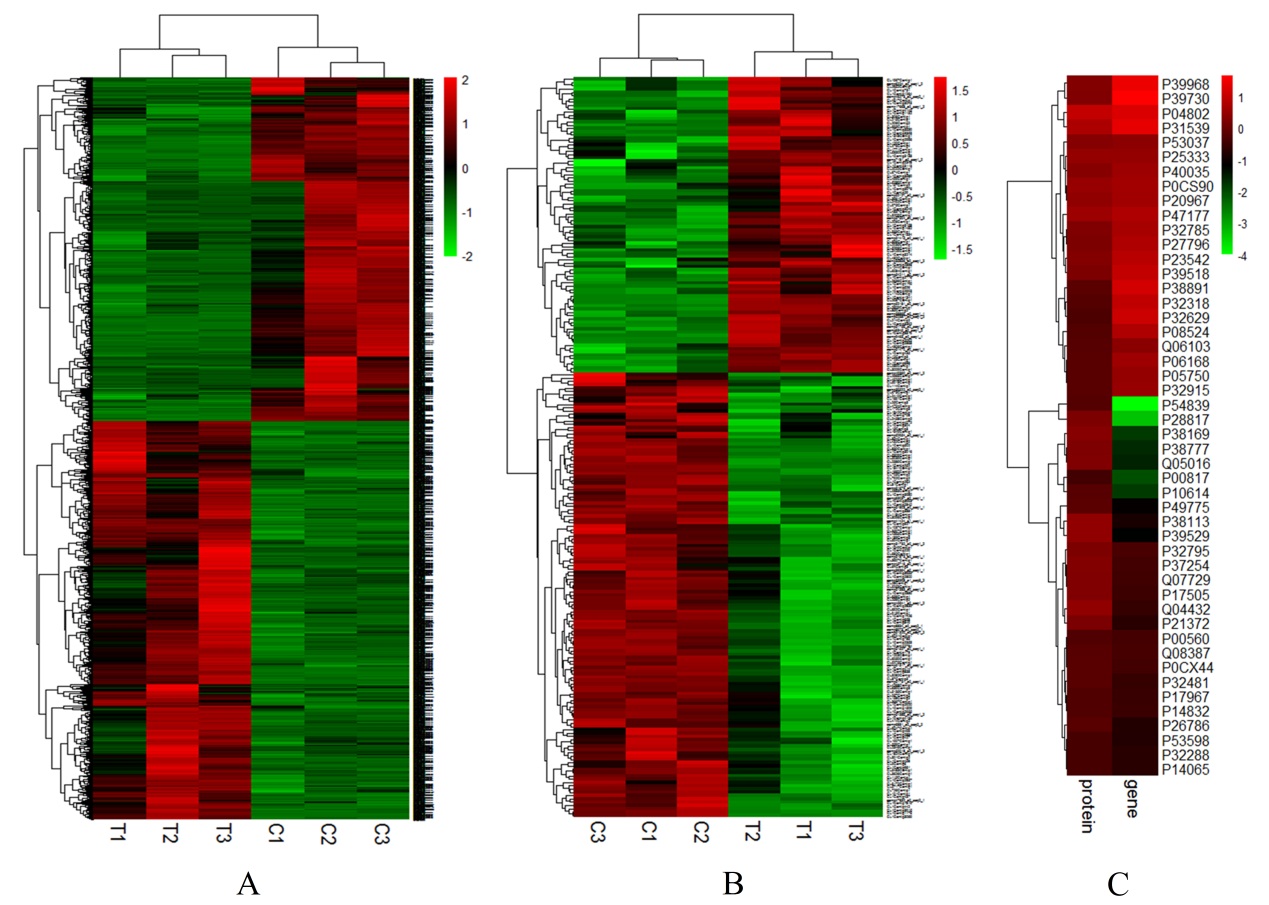


**Figure S5.** Heatmap analyses of the correlation of three biological replicates in transcriptome (A) and in proteome (B), and of the correlation of DEPs and DEGs (C). The small panel with color gradient represents the changes of protein abundance or mRNA abundance from down-regulated (green) to up-regulated (red).


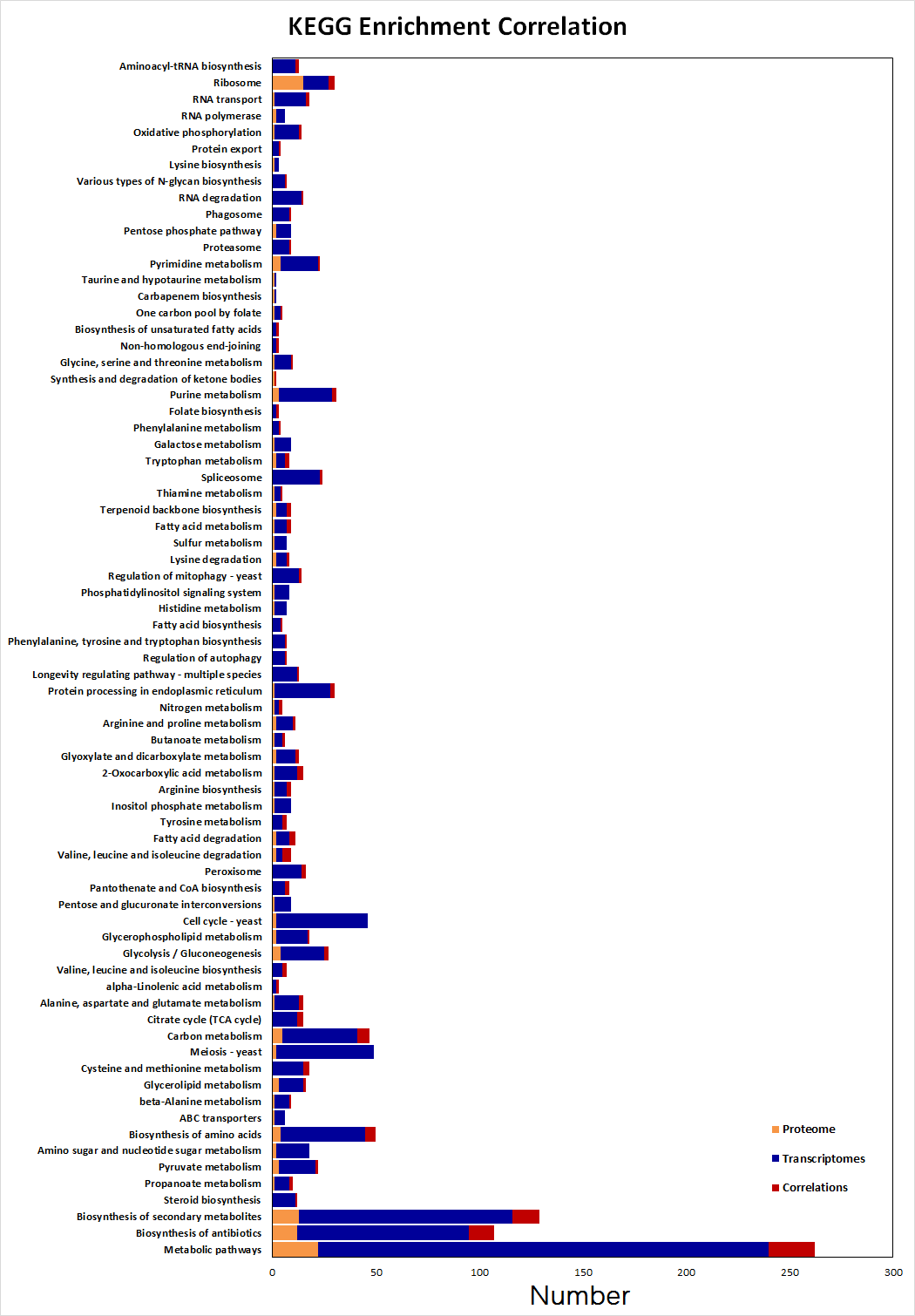


**Figure S6.** Number of KEGG enrichment correlation between proteome and transcriptome.


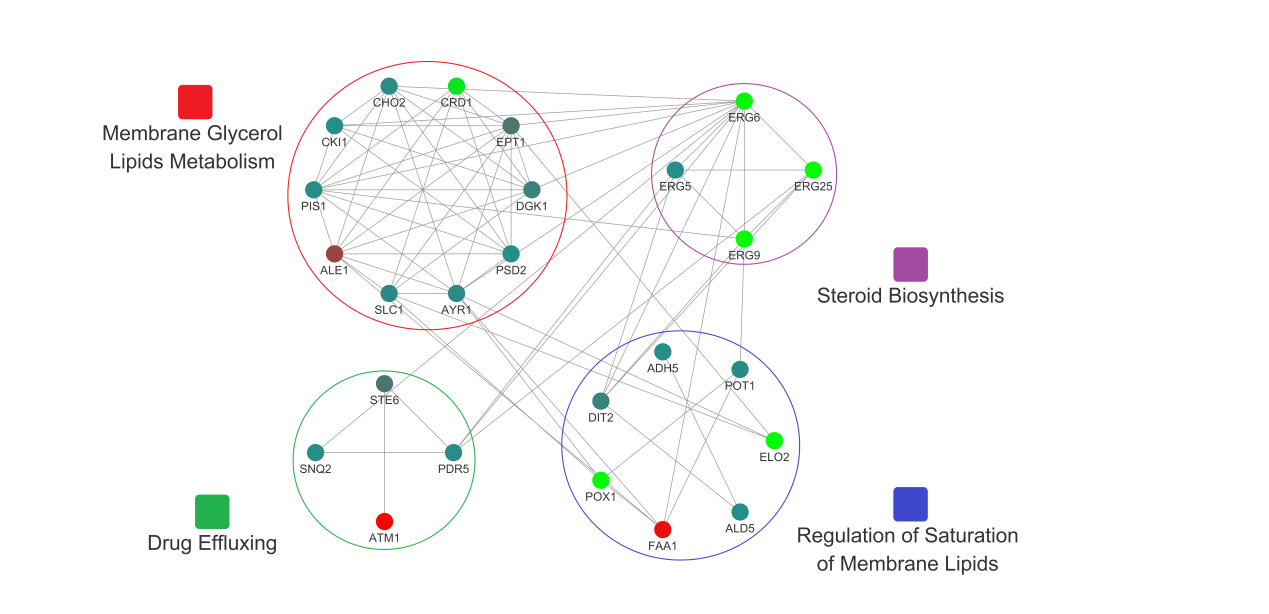


**Figure S7.** Protein-protein interaction network of *T. roseum* under cuminal exposure compared with the control. The proteins encoded by DEGs functioning in the fungal membrane composition and drug effluxing were selected. Interactions are shown by the lines connecting each node based on available evidence in the database. Interactions are integrated into different metabolic sub-blocks underpinning the fungal membrane composition and drug effluxing and different sub-blocks are indicated by the colored circles.


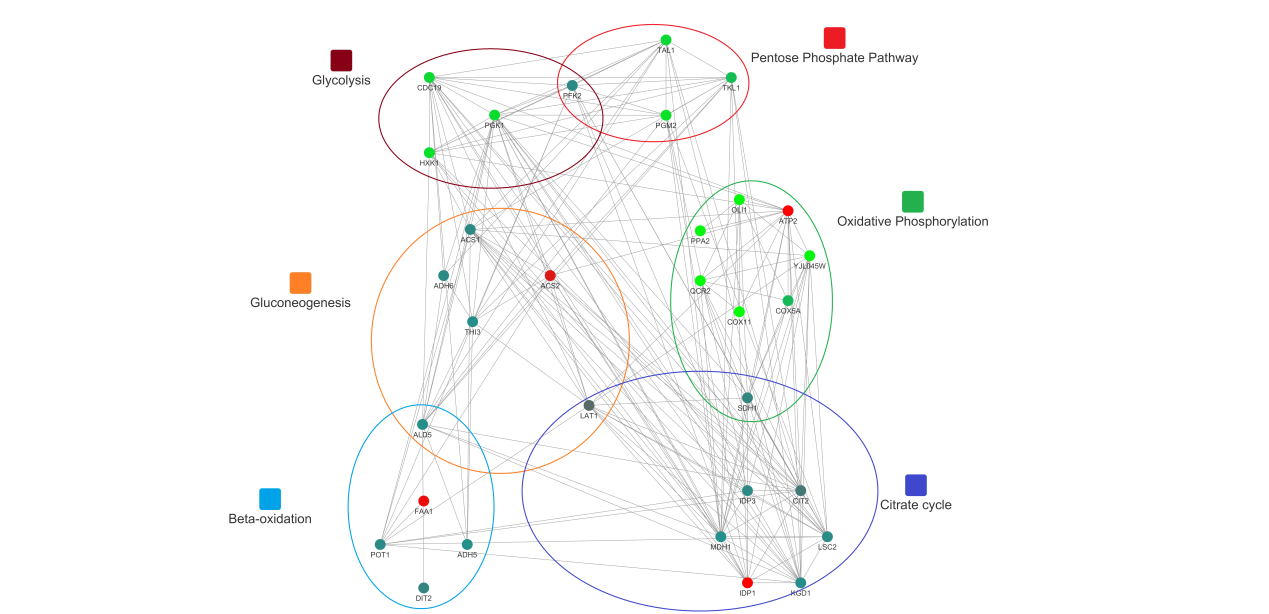


**Figure S8.** Protein-protein interaction network of *T. roseum* under cuminal exposure compared with the control. The proteins encoded by DEGs functioning in the fungal carbohydrate and energy metabolism were selected. Interactions are shown by the lines connecting each node based on available evidence in the database. Interactions are integrated into different metabolic sub-blocks underpinning the fungal carbohydrate and energy metabolism and different sub-blocks are indicated by the colored circles.
